# Establishing Host–Virus Link Through Host Metabolism: Viral DNA SIP Validation Using T4 Bacteriophage and *E. coli*

**DOI:** 10.1007/s00284-024-03774-x

**Published:** 2024-07-14

**Authors:** Vuong Quoc Hoang Ngo, Maximilien Sotomski, Angeline Guenne, Mahendra Mariadassou, Mart Krupovic, François Enault, Ariane Bize

**Affiliations:** 1 Université Paris-Saclay, INRAE, PROSE, 92761 Antony, France; 2 Université Paris-Saclay, INRAE, MaIAGE, 78350 Jouy-en-Josas, France; 3 BioinfOmics, MIGALE Bioinformatics Facility, Université Paris-Saclay, INRAE, 78350 Jouy-en-Josas, France; 4 CNRS UMR6047, Archaeal Virology Unit, Institut Pasteur, Université de Paris, 75015 Paris, France; 5 Université Clermont Auvergne, CNRS, LMGE, 63000 Clermont-Ferrand, France

## Abstract

DNA Stable Isotope Probing is emerging as a potent methodology for investigating host–virus interactions, based on the essential reliance of viruses on host organisms for the production of virions. Despite the anticipated link between host isotopic compositions and the generated virions, the application of stable isotope probing to viral DNA has never been evaluated on simple biological models. In this study, we assessed the efficacy of this method on the bacteriophage T4 and its host, *Escherichia coli*. Through the cultivation of *E. coli* cells on a ^13^C-enriched substrate and subsequent propagation of T4 bacteriophage, we examine the degree of isotopic enrichment in viral DNA. Our investigation reveals a strong correlation between the proportion of ^13^C_6_-d-glucose in the growth substrate and the buoyant density in CsCl gradient of T4 DNA, confirming the validity of DNA SIP in viral ecology. These findings underscore the potential of DNA SIP as a robust tool for characterizing the diversity of viruses infecting hosts with specific metabolic activities and provide then a foundation for further exploration in viral ecology research.

## Introduction

Stable Isotope Probing (SIP) [1] has been recently employed in viral ecology to identify viruses infecting hosts with specific metabolism (e.g. [2–7]), such as ammonia oxidation [3], methane oxidation [4], or methanogenesis [6]. This method is gaining recognition as a promising approach due to the inherent dependency of all viruses on host resources for virion production, suggesting a potential association between host isotopic composition and the isotopic signature of produced virions. This has been previously shown by NanoSIMS on simple biological models, namely cyanobacterial and eukaryotic algal–virus systems [7]. NanoSIMS is generally employed to acquire a map of the elemental and isotopic composition of a sample, at the nanoscale. In this previous study [7], hosts were grown on ^13^C- or ^15^N-labeled substrates and individual viral particles enriched in these isotopes were visualized by NanoSIMS, showing the transfer from the hosts to the viruses.

In contrast, DNA SIP for the study of host-virus interactions has been directly applied to microbial communities [2–5, 8], without validation on simple systems. DNA SIP relies on the use of a growth substrate labeled with a rare and stable isotope, such as ^13^C or ^15^N. After their extraction, the DNA fragments are separated by ultracentrifugation in caesium chloride (CsCl) gradient according to their buoyant density. The labeled DNA fragments, denser, can thereby be recovered and specifically characterized with classical molecular biology techniques, such as sequencing. In the few studies where DNA SIP was employed to decipher host–virus relationships, either total DNA [3, 4, 8] or cellular DNA [6] has been used. In principle, the application of SIP to viral DNA alone should be feasible with sufficient material, potentially enhancing the detection sensitivity for a broader diversity of labeled viruses. Indeed, when DNA stable isotope probing is applied to the total DNA, cellular DNA is by far dominant, likely preventing the detection of many low abundant viruses. Here, we validate viral DNA SIP on a simple biological model, using T4 bacteriophage DNA obtained by propagation on *Escherichia coli* cells. We therefore also provide some reference profiles of viral DNA density. Viral DNA SIP could be used in viral ecology to identify the range of viruses infecting hosts with a specific metabolism, with high sensitivity.

## Materials and Methods

### Culture Conditions and Virion Preparation

*E. coli* strain B (DSM 613) cells were grown during 30 h at 37 °C, under 400-rpm agitation, in 20 mL of M9 liquid minimal medium [M9, Minimal salts, 5X (Sigma-Aldrich), MgSO_4_ (1 mM), CaCl_2_ (0.3 mM), and d-glucose (10% m:v)], inoculated with 20 µL of an overnight culture in LB medium (Fisher Bioreagents, 25 g/L). The glucose was a mix of ^13^C-labeled (d-Glucose-13C6, 99% ^13^C, Cortecnet) and non-labeled (Sigma-Aldrich) d-glucose in various proportions, ranging from 0 to 100%, in order to evaluate the efficiency and the sensitivity of the proposed approach.

For infection with T4 (DSM 4505), the same conditions were employed except that minimal medium was supplemented with 20 µL of CaCl_2_ (0.5 M) and MgCl_2_ (1 M) solutions and T4 virions were added before incubation at a multiplicity of infection (MOI) of ~ 10^–7^. After 30 h of cultures were centrifuged at 5 000 g for 15 min at 10 °C. The supernatants were filtered at 0.22-µm pore size with PES filters (FisherBrand). The obtained virion suspensions were stored at 4 °C until further use. A low MOI combined with a long incubation time was employed, both to limit the input of unlabeled carbon and enable the occurrence of several viral infection cycles, resulting in the production of a high number of virions.

### Isotope Ratio Mass Spectrometry

Non-infected cells were obtained as described in Sect. “Culture Conditions and Virion Preparation”. They were washed once in PBS and dried overnight at 55 °C. They were analyzed by EA-IRMS with FlashEA 1112 Series and a Delta V Plus (Thermo Fisher Scientific), as previously described [9], using 547 ± 171 µg of cells.

### DNA Extraction

For T4 virions obtained as described in Sect. “Culture Conditions and Virion Preparation”, T4 DNA was extracted according to the following procedure. Virions were firstly concentrated, either using Amicon Ultra-15 centrifugal Filter units (Meck Millipore) or by centrifugation at 20,000×*g* for 4 h at 4 °C, followed by pellet suspension in 1-mL supernatant. The concentrated T4 suspensions were incubated with 10-µL DNase (DNase I THERMO, 1 Unite/µL) at room temperature for 20 min. The DNase was inactivated at 75 °C for 5 min. Viral DNA was subsequently extracted with the Phage DNA Isolation Kit (Norgen), with the following minor modifications. For proteinase treatment (proteinase K from *Tritirachium album*, Sigma-Aldrich, 20 mg/mL), samples were incubated for 15 min at 55 °C with 80 µL of virion suspension. For the final elution, either 2 elutions with 75 µL, or 3 elutions with 50 µL, elution buffer, were performed.

All DNAs were quantified with a Qubit fluorometer and the dsDNA HS kit (Thermo Fisher Scientific).

### Separation of DNA in an Isopycnic Gradient

DNAs were separated according to their density by ultracentrifugation in an isopycnic CsCl gradient, as previously described [10]. Briefly, between 200 ng and 2 µg of DNA were added to a solution of CsCl and Tris–EDTA in each ultracentrifuge tube, to reach an average density of 1.725 g/mL. The 2-mL Quick-Seal polyallomer tubes were centrifuged during 20 h at 120 000×*g* and 20 °C, using a TLA-120.2 rotor (Beckman). After ultracentrifugation, fractions of 100 μL were recovered from the bottom of each tube by pumping water into the top of the tube with a constant flow (200 μL/min). The density of each fraction was assessed by measuring its refractive index (Reichert Arias 500 refractometer). The DNA concentrations were determined with the Qubit dsDNA HS Assay Kit, according to the manufacturer’s instructions.

The raw data are provided as Supplementary Information. The linear regression was calculated with the lm function from R 4.3.1 in R studio (2023.12.0 + 369).

## Results and Discussion

### The ^13^C Enrichment of Uninfected *E. coli* Cells is Consistent with the Isotopic Composition of the Growth Substrate

**Fig. 1:**
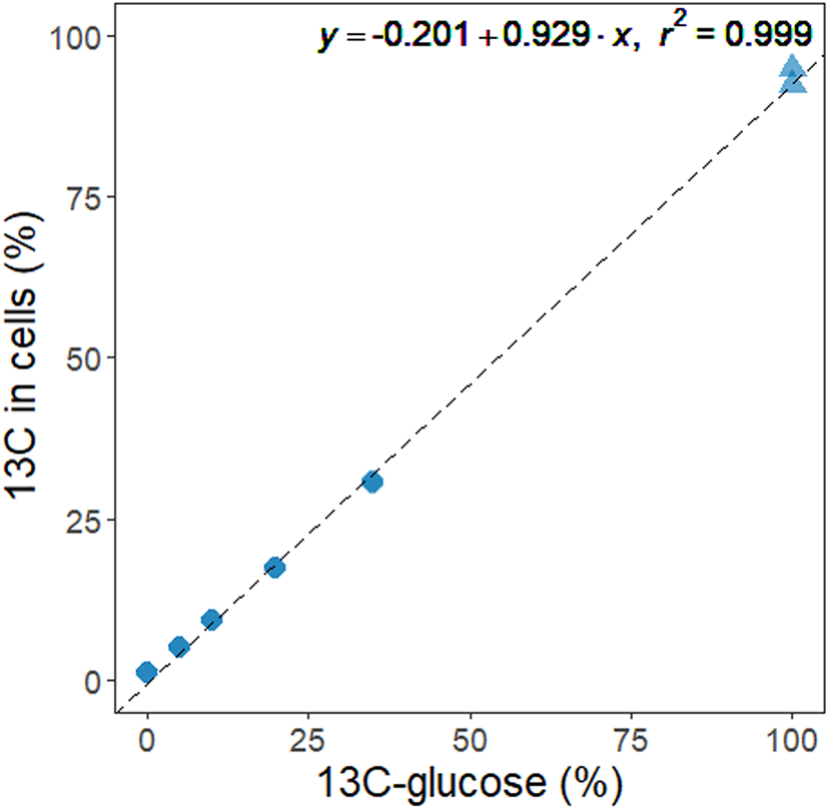
Isotopic composition of *E. coli* cells, as determined by EA-IRMS, in function of the percentage of ^13^C_6_-d-glucose employed in the d-glucose substrate. There are 4 replicates by condition. The different shapes, circles, and triangles represent two distinct experimental series. The dotted line and the equation shown in the plot are related to the linear regression

As a control, we first measured the isotopic composition of uninfected *E. coli* cells grown in minimal medium, by EA-IRMS (Fig. 1). The sole carbon source was d-glucose containing various proportions of ^13^C_6_-d-glucose. Overall, the measured ^13^C content of cells was slightly inferior to the theoretical ^13^C content of the substrate, with the relative difference varying between − 13.55 and 1.40%. Such difference cannot be explained by the addition of the inoculum, which contained LB medium, because a 1000× dilution was applied for inoculation. It could rather result from the ^13^C content of ^13^C_6_-d-glucose being slightly inferior to 100% (≥ 99% according to the supplier), combined with biases introduced during medium preparation and IRMS measurement. Despite these minor differences, a very strong correlation was obtained between the ^13^C content of substrate and cells, as expected (Fig. 1, *R*^2^ = 0.999). The replicates (*N* = 2) showed high reproducibility.

### The buoyant Density of T4 DNA Correlates with the Isotopic Composition of the Substrate Used for Host Growth

**Fig. 2:**
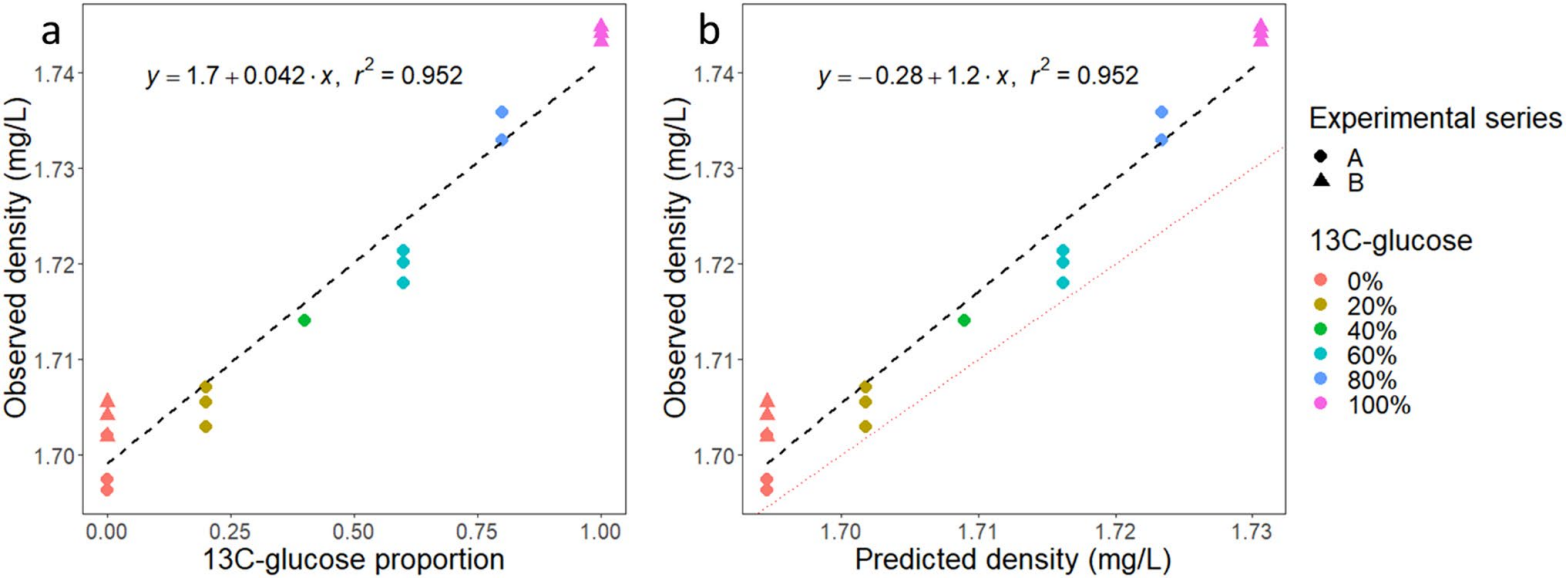
Buoyant density of T4 bacteriophage DNA measured in CsCl gradient. **a**T4 DNA density as a function of the proportion of ^13^C_6_-d-glucose employed in the d-glucose substrate for host growth. **b** T4 DNA density as a function of the theoretical density calculated according to the equation presented in the text [10]. The identity line is shown with red dots. The dashed line and equations shown on the plots are related to the linear regressions. The data originate from 2 distinct experimental series. In experimental series A, 0% and 100% ^13^C_6_-d-glucose proportions were tested, in biological triplicates. In experimental series B, 0%, 20%, 40%, 60%, and 80% ^13^C_6_-d-glucose proportions were tested, in biological triplicates. However, there was an experimental problem for one replicate of the 40% condition and one of the 60% condition, so that there are only duplicates shown in these cases

Subsequently, *E. coli* cells grown on minimal medium with various proportions of ^13^C_6_-d-glucose were infected by T4 bacteriophage. T4 DNA was extracted and separated on a CsCl gradient. A good reproducibility was observed among replicates, and small differences are visible across the two different experimental series. Overall, a strong correlation was obtained between the observed T4 DNA densities and the percentages of ^13^C_6_-d-glucose in the substrate (Fig. 2b, *R*^2^ = 0.952), resulting from the link between the isotopic composition of the host and the produced virions. Since we observed a correlation between the ^13^C content of the growth substrate and the isotopic composition of both the cells and T4 DNA, we can conclude that there is a correlation between the isotopic composition of the host and the virions they produce, as expected, although the correlation coefficient was not directly measured.

### Observed T4 DNA Densities Are Greater than Expected from Empirical Models

We calculated the theoretical expected density for T4 DNA, by relying on a previously established empirical formula [11]:

$$\rho_{exp} = 1.660 + \left( {0.098 \times \left[ {G + C} \right]} \right) + 0.036 \times \left[ {{}^{13}C} \right],$$

where $\left[G+C\right]$ is the GC content of the considered DNA (0.3530 for T4, NC_000866.4) and [^13^*C*] is its ^13^C content.

The obtained predicted densities were significantly lower than the measured ones (Fig. 2b). T4 DNA contains glucosylated hydroxymethylcytosine (HmC) instead of cytosine [12], affecting its buoyant density: it was detected as heavier than expected without DNA modification, in a previous study [13], with a value of 1.698 compared to 1.694 according to the above formula. In the present study, an average value of 1.701 (± 0.004 STD) was obtained for unlabeled T4 DNA, based on duplicates. For the fully labeled T4 DNA, the average density value was of 1.744, compared to an expected value of 1.731 according to the empirical model. Based on the linear regression from Fig. 2a, fully label T4 DNA has a density increase of 0.042 (compared to 0.036 in the above model). Models linking the GC content to the buoyant density would thus need to be adjusted for modified DNAs. Here, we do not suggest a new model, as it would require the analysis of DNA presenting the same modifications as T4, but with various GC contents.

### Viral DNA SIP: Powerful Tool to Establish the Link Between Virus and Its Host

Several experimental methods are already available to identify hosts of viruses, including digital droplet PCR [14], proximity ligation [15], epicPCR [16], or viral tagging [17]. To our knowledge, none of these methods are trivial to implement, each possessing distinct advantages and limitations. While some methods are targeted, such as those based on PCR, others require the cultivation of hosts, as is the case with viral tagging. Proximity ligation, conversely, is untargeted but necessitates meticulous design and data treatment to mitigate noise.

Experimental techniques utilizing stable isotopes, such as DNA Stable Isotope Probing (DNA SIP) or NanoSIMS, complement the aforementioned approaches in host–virus interaction studies, particularly as they relate to metabolic activity. This aspect is crucial for functional ecology, with these methods showing significant promise in elucidating the connections between viruses and major biogeochemical cycles. They are thus worth further developing and investigating. As mentioned in the introduction, DNA SIP has been successfully applied in viral ecology studies, using either cellular DNA [3, 4, 8] or total DNA [6]. We postulate that applying SIP to viral DNA separately, although challenging, would be complementary and would provide a different view on the viral diversity, with increased sensitivity and, likely, a better detection of purely virulent viruses. Compared to viral DNA, cellular DNA or total DNA is indeed expected to be enriched in temperate virus sequences, present in cellular genomes. Moreover, since cellular DNA is dominant in the total DNA, one can expect a lower sensitivity for detecting DNA from low abundant, purely virulent viruses, compared to an approach focusing on the DNA directly extracted from virions.

However, in viral DNA SIP that targets a specific metabolism, only a limited proportion of the microbial community is likely to be labeled, which poses a notable challenge. This technique requires a considerable amount of environmental viral DNA for analysis. Based on our experimental data, at least 400 ng of viral DNA from an environmental sample is required to reliably discriminate between the ^12^C and ^13^C buoyant density peaks. This requirement highlights the importance of careful experimental design and emphasizes the need for efficient viral DNA extraction and concentration techniques to meet the threshold for successful isotopic discrimination.

## Conclusion

In this study, using a simple biological model, we observed a very strong correlation between the proportion of ^13^C_6_-d-glucose in the substrate and T4 DNA buoyant density. We therefore validate for the first time the use of viral DNA SIP on a model system. Similar to DNA SIP applied to environmental microbial communities, this approach could be used specifically in viral ecology to identify, with a high level of sensitivity, the range of viruses infecting hosts with a specific metabolism.
